# Prognostic value of bedside lung ultrasound score in patients with COVID-19

**DOI:** 10.1186/s13054-020-03416-1

**Published:** 2020-12-22

**Authors:** Li Ji, Chunyan Cao, Ying Gao, Wen Zhang, Yuji Xie, Yilian Duan, Shuangshuang Kong, Manjie You, Rong Ma, Lili Jiang, Jie Liu, Zhenxing Sun, Ziming Zhang, Jing Wang, Yali Yang, Qing Lv, Li Zhang, Yuman Li, Jinxiang Zhang, Mingxing Xie

**Affiliations:** 1 Department of Ultrasound, Union Hospital, Tongji Medical College, Huazhong University of Science and Technology, 1277# Jiefang Ave, Wuhan, 430022 China; 2 Hubei Province Key Laboratory of Molecular Imaging, 1277# Jiefang Ave, Wuhan, 430022 China; 3 Department of Emergency Surgery, Union Hospital, Tongji Medical College, Huazhong University of Science and Technology, 1277# Jiefang Ave, Wuhan, 430022 China

**Keywords:** COVID-19, Lung ultrasound, LUS score, Acute respiratory distress syndrome (ARDS), Prognosis

## Abstract

**Background:**

Bedside lung ultrasound (LUS) has emerged as a useful and non-invasive tool to detect lung involvement and monitor changes in patients with coronavirus disease 2019 (COVID-19). However, the clinical significance of the LUS score in patients with COVID-19 remains unknown. We aimed to investigate the prognostic value of the LUS score in patients with COVID-19.

**Method:**

The LUS protocol consisted of 12 scanning zones and was performed in 280 consecutive patients with COVID-19. The LUS score based on B-lines, lung consolidation and pleural line abnormalities was evaluated.

**Results:**

The median time from admission to LUS examinations was 7 days (interquartile range [IQR] 3–10). Patients in the highest LUS score group were more likely to have a lower lymphocyte percentage (LYM%); higher levels of D-dimer, C-reactive protein, hypersensitive troponin I and creatine kinase muscle-brain; more invasive mechanical ventilation therapy; higher incidence of ARDS; and higher mortality than patients in the lowest LUS score group. After a median follow-up of 14 days [IQR, 10–20 days], 37 patients developed ARDS, and 13 died. Patients with adverse outcomes presented a higher rate of bilateral involvement; more involved zones and B-lines, pleural line abnormalities and consolidation; and a higher LUS score than event-free survivors. The Cox models adding the LUS score as a continuous variable (hazard ratio [HR]: 1.05, 95% confidence intervals [CI] 1.02 ~ 1.08; P < 0.001; Akaike information criterion [AIC] = 272; C-index = 0.903) or as a categorical variable (HR 10.76, 95% CI 2.75 ~ 42.05; P = 0.001; AIC = 272; C-index = 0.902) were found to predict poor outcomes more accurately than the basic model (AIC = 286; C-index = 0.866). An LUS score cut-off > 12 predicted adverse outcomes with a specificity and sensitivity of 90.5% and 91.9%, respectively.

**Conclusions:**

The LUS score devised by our group performs well at predicting adverse outcomes in patients with COVID-19 and is important for risk stratification in COVID-19 patients.

## Background

Coronavirus disease 2019 (COVID-19) caused by severe acute respiratory syndrome coronavirus 2 (SARS-CoV-2) has become a global threat, resulting in severe illnesses such as acute respiratory distress syndrome (ARDS), multi-organ dysfunction syndrome and even mortality [1]. Since there is no specific medicine to cure COVID-19, supportive care is the major treatment during hospitalization [2]. Close follow-up and medicine to relieve symptoms are sufficient for non-critically ill patients, while for severe and critically ill patients, aggressive treatment and admission to intensive care unit (ICU) are needed. However, many non-critically ill patients at admission may deteriorate suddenly during hospitalization [3]. Consequently, early prediction of disease progression may be fundamental in delivering appropriate health care for COVID-19 patients. Several demographic and clinical parameters have been recently shown to have some value for risk stratification in the development of the disease [4–7]. However, COVID-19 is a kind of respiratory disease, and the lungs are the major organ affected [8]. Therefore, quantitative imaging data regarding lung lesions may be essential for in-hospital care to aid in identifying those who may benefit from more intensive monitoring and treatment.

Lung ultrasound (LUS) imaging is a fast, non-invasive, sensitive and quantitative tool to assess multiple pulmonary pathologies, such as pulmonary oedema, pneumonia and interstitial lung disease [9–11]. More recently, LUS has also been used to detect lung involvement and monitor changes in patients with COVID-19, especially children and pregnant women [12, 13]. Indeed, ultrasound is the sole imaging modality with accessibility to the bedside of patients for timely identification of pulmonary and other organ complications, reducing the risk of contagiousness and the need to move unstable patients [14]. Recent studies have shown that LUS is an independent predictor of adverse outcomes in patients with pulmonary disease [15, 16]; however, the prognostic significance of LUS in patients with COVID-19 is unclear. Therefore, the purpose of this study was to investigate whether the LUS score at admission was independently predictive of poor outcomes in patients with COVID-19.

## Methods

### Study design and population

This was a prospective, single-centre, observational study that included 280 consecutive patients from the designated hospital to treat COVID-19 patients, the west and tumour branch of Union Hospital, Tongji Medical College, Huazhong University of Science and Technology, from January 21, 2020, to March 10, 2020. Inclusion criteria consisted of COVID-19 diagnosis according to the interim guidance of the World Health Organization [17], with age > 18 years. The exclusion criteria were as follows: heart failure, interstitial pneumonia, tuberculosis, bronchiectasis, chronic obstructive pulmonary disease (COPD), other pulmonary disease hampering image acquisition (significant pleural effusion, previous pneumonectomy, breast prosthesis) or suboptimal ultrasound window.

The study complied with the edicts of the 1975 Declaration of Helsinki [18] and was approved by the institutional ethics board of Union Hospital Tongji Medical College, Huazhong University of Science and Technology. Written informed consent was waived for all participants with emerging infectious diseases.

### Clinical data and outcomes

Patients’ demographic characteristics, symptoms, laboratory tests, comorbidities, complications, treatment and outcomes were extracted from electronic medical records by a single investigator. Laboratory tests were recorded only if they were obtained within 7 days of admission. These data were independently reviewed and entered into the computer database by two analysts (L.L.J. and R.M.).

The outcomes included: (1) in-hospital mortality; (2) ARDS. The primary endpoint of the study was in-hospital mortality, and ARDS was the secondary endpoint. All prespecified outcomes were confirmed through patient electronic medical records and evaluated by two experienced investigators (J.L. and M.J.Y.) who were blinded to the ultrasound data. ARDS was defined according to the Berlin Definition [19]. The final follow-up date was March 25, 2020.

### Lung ultrasound imaging protocol and analysis

Lung ultrasound examinations were performed by trained sonographers (Y.L.D., W.Z., S.S.K., Z.M.Z. and Z.X.S.) using portable ultrasound equipment (Mindray M7, M8, M9 and GE Logiq E9) with a 1–6 MHz convex transducer. The unilateral lung was divided into anterior, lateral and posterior fields using anterior and posterior axillary lines, and each field was divided into superior and inferior areas using two axial lines (one above the diaphragm and the other 1 cm above the nipples). A total of 12 regions were assessed using a two-dimensional view with the probe placed perpendicular to the chest wall and evaluated for the following signs: pleural line (a horizontal hyperechoic line between the ribs), A-lines (horizontal reverberation artefacts repeated at a constant distance equal to the distance between pleural line and probe surface), B-lines (vertical hyperechoic reverberation artefacts deriving from the pleural line) and consolidation (presence of a tissue-like pattern) [20, 21].

**Fig. 1:**
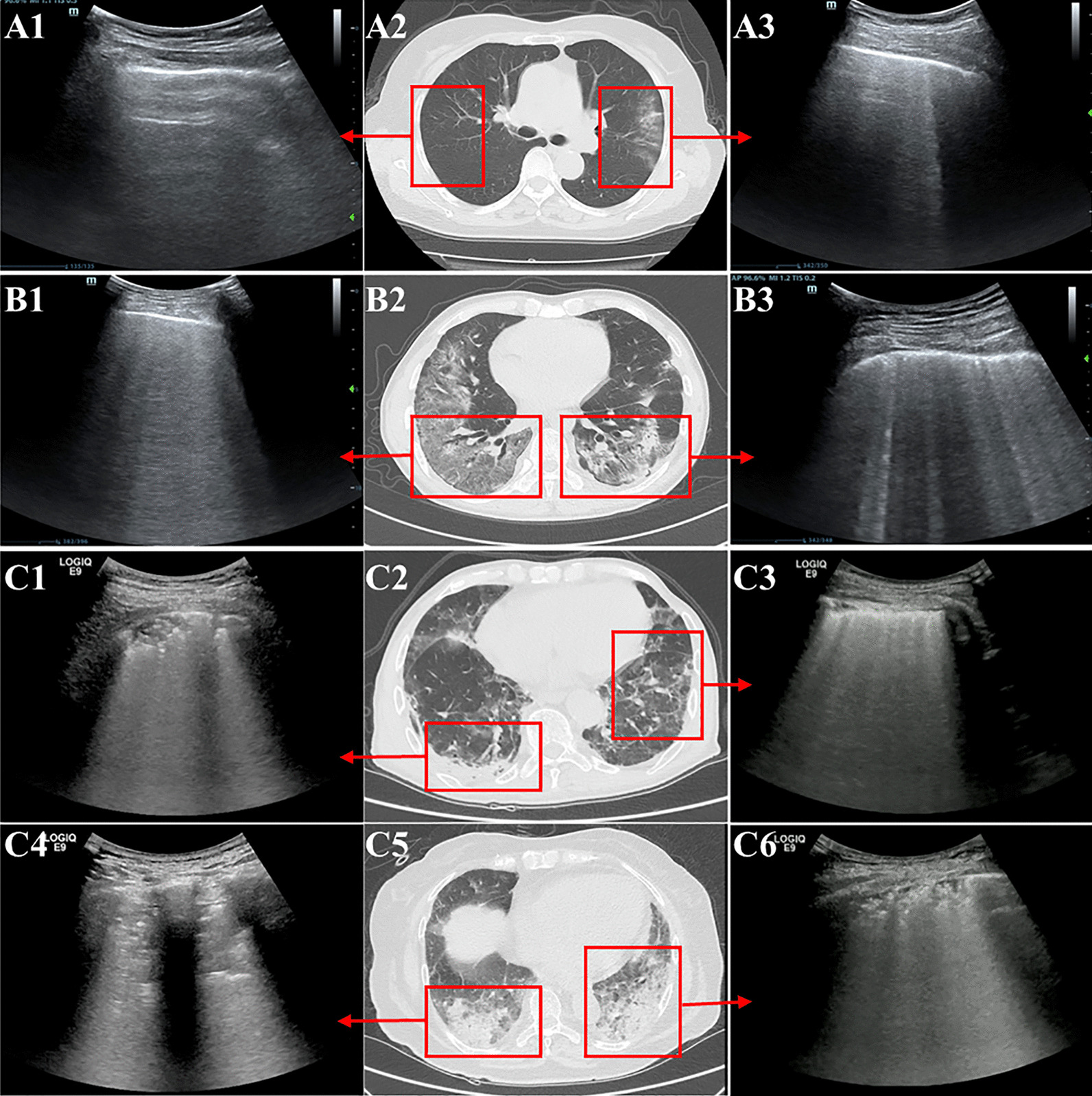
Typical lung ultrasound and corresponding lung CT images of patients with various LUS score. A: Lung images of a patient with a low LUS score; A1: normal pleural line and no B-line, score 0; A3: normal pleural line and well-spaced B-lines ≥ 3, score 1; A2: lung CT in the corresponding areas. B: Lung images of a patient with a moderate LUS score; B1: normal pleural line and multiple coalescent B-lines, score 2; B3: irregular pleural line and well-spaced B-lines ≥ 3, score 2; B2: lung CT in the corresponding areas. C: Lung images of a patient with a high LUS score; C1: irregular pleural line and lung consolidation, score 4; C3: blurred pleural line and multiple coalescent B-lines, score 4; C4: blurred pleural line and lung consolidation, score 5; C6: irregular pleural line and lung consolidation, score 4; C2, C5: lung CT in the corresponding areas. (Note: Lung images of all 12 regions of each patient are not shown in the figure. The total LUS score should be calculated with a total score of 12 regions.)

Offline image analysis was performed by two investigators (L.J. and C.Y.C.) with experience in LUS who were blinded to the clinical data and other radiologic features. Images were evaluated independently. After separate evaluations, final decisions were reached by consensus. In each region, LUS signs including B-lines/consolidation and pleural line abnormalities were assessed, and the worst ultrasound pattern was recorded. B-lines/consolidation were quantitatively scored according to a previous study [21]: (1) score 0: well-spaced B-lines < 3; (2) score 1: well-spaced B-lines ≥ 3; (3) score 2: multiple coalescent B-lines; (4) score 3: lung consolidation. The pleural line was quantitatively scored as follows: (1) score 0: normal; (2) score 1: irregular pleural line; (3) score 2: blurred pleural line. A composite sore of each region was calculated by summing the individual scores for B-lines/consolidation (score 0–3) and pleural abnormalities (score 0–2). The sum of the scores in all twelve zones yielded a final score of the COVID-19 patient (ranging from 0 to 60), defined as the LUS score. Typical lung ultrasound and corresponding lung computerized tomography (CT) images of patients with various LUS score are shown in Fig. 1.

### Statistical analysis

Continuous variables are expressed as the mean ± SD or median (interquartile range [IQR]), as appropriate. Categorical variables are presented as frequencies (percentages). Continuous variables were compared using analysis of variance (ANOVA) for normally distributed data or the Kruskal-Wallis test for non-normally distributed data. Categorical variables were compared using the chi-square test or Fisher’s exact test. Estimations of the predictor of adverse events were performed using univariate and multivariate Cox regression models. All potential predictors of adverse outcomes were entered into univariate analyses. Variables with P < 0.001 in univariate analysis were entered into multivariate Cox regression models. For multivariable analysis, a separate model including clinical variables and LUS score was used to determine the independent predictors of poor outcome. Model performance was assessed using the Akaike information criterion (AIC) and the C-index. Receiver operator curve (ROC) analysis was performed to examine the sensitivity and specificity of prognosis parameters for adverse events and to determine the best cut-off value (maximum Youden index) for predicting future events. Kaplan–Meier curves were used to examine cumulative event rates, and differences between groups were tested using the log rank test. A two-sided value of *P* < 0.05 was considered significant. Statistical analyses were performed using SPSS version 22.0 (SPSS Inc, Chicago, Illinois), R-language 4.0.1 and MedCalc version 19.0.7 (MedCalc Software, Ostend, Belgium).

## Results

### Clinical characteristics

A total of 280 patients with COVID-19 who met the inclusion criteria were identified (age, 55 years [IQR, 40–65 years]; gender, 141 male), including 153 (54.6%) with low LUS score, 70 (25%) with moderate LUS score and 57 (20.4%) with high LUS score. Table 1 summarizes the baseline clinical characteristics of the patients stratified by the level (low, moderate, high) of the LUS score. Patients in the high LUS score group were older and had a significantly higher incidence of comorbidities (including hypertension, diabetes, chronic cardiovascular disease and malignancy), lower LYM% and SO_2_%, higher levels of CRP, D-dimer, hs-TnI and CK-MB, and lower oxygenation index than patients in the low and moderate LUS score groups. There were no significant differences in gender, BMI, respiratory rate at admission or the prevalence of chronic liver disease in patients with COVID-19 among the low, moderate and high LUS score groups. More patients with higher LUS score were treated with medicines (antiviral, antibiotic and glucocorticoid) and high-flow oxygen than those with lower LUS score. Only patients with high LUS score received invasive mechanical ventilation (n = 17) therapy and admission to the ICU (n = 17).

**Table 1:** Baseline characteristics of patients with COVID-19 according to LUS score

Variables	Total population (n = 280)	LUS score			p value
		Low 0–1 (n = 153)	Moderate 2–12 (n = 70)	High > 12 (n = 57)	
*Demographic data*					
Age, year	55 [40 ~ 65]	46 [34 ~ 57]	59 [48 ~ 68]	69 [60 ~ 76]	< 0.001
Male, n (%)	141 (50.4)	73 (47.7)	37 (52.9)	31 (54.4)	0.632
BMI, Kg/m [2]	23.11 ± 3.31	23.24 ± 3.27	23.29 ± 3.08	23.16 ± 3.27	0.957
Temperature, ℃	38.4 [37.8 ~ 38.9]	38.0 [37.8 ~ 38.8]	38.4 [37.8 ~ 38.7]	38.6 [38.0 ~ 39.0]	0.042
Respiratory rate, min^−1^	20 [19 ~ 20]	20 [18 ~ 20]	20 [18.5 ~ 20]	20 [19 ~ 21]	0.207
SaO2 (%)	98 [97 ~ 99]	99 [98 ~ 99]	99 [98 ~ 99]	98 [95 ~ 99]	0.014
*Comorbidities, n (%)*	85 (30.4)	25 (16.3)	21 (30.0)	39 (68.4)	< 0.001
Hypertension, n (%)	58 (20.7)	16 (10.5)	15 (21.4)	27 (47.4)	< 0.001
Diabetes, n (%)	27 (9.6)	4 (2.6)	6 (8.6)	17 (29.8)	< 0.001
Chronic cardiovascular disease, n (%)	24 (8.6)	6 (3.9)	0 (0.0)	18 (31.6)	< 0.001
Chronic liver diseases, n (%)	11 (3.9)	4 (2.6)	2 (2.9)	5 (8.8)	0.161
Malignancy, n (%)	13 (4.6)	2 (1.3)	2 (2.9)	9 (15.8)	< 0.001
*Laboratory results*					
Lymphocytes %	24.29 ± 10.88	29.18 ± 8.43	27.55 ± 8.79	14.60 ± 9.32	< 0.001
CRP, mg/L	6.30 [2.00 ~ 40.13]	2.49 [0.81 ~ 8.49]	3.40 [1.47 ~ 10.69]	27.43 [4.18 ~ 66.41]	< 0.001
D-dimer, ug/L	0.99 [0.37 ~ 3.70]	0.27 [0.20 ~ 0.94]	0.34 [0.25 ~ 1.20]	1.65 [0.85 ~ 6.52]	< 0.001
hs-TnI, ng/L	2.6 [1.4 ~ 2.6]	2.2 [1.4 ~ 2.8]	1.7 [0.9 ~ 5.3]	18.3 [2.5 ~ 113.2]	0.001
CK-MB, U/L	0.5 [0.3 ~ 6.0]	0.4 [0.3 ~ 0.6]	0.4 [0.3 ~ 0.8]	7.0 [0.4 ~ 13.5]	0.001
PaO_2_:FiO_2_, mmHg					
> 300	246 (87.9)	153 (100.0)	68 (97.1)	25 (43.9)	< 0.001
200–300	22 (7.9)	0 (0.0)	2 (2.9)	20 (35.1)	< 0.001
≤ 200	12 (4.3)	0 (0.0)	0 (0.0)	12 (21.1)	< 0.001
*Treatments*					
Antiviral therapy, n (%)	82 (29.3)	19 (12.4)	28 (40.0)	35 (61.4)	< 0.001
Antibiotic therapy, n (%)	78 (27.9)	18 (11.8)	26 (37.1)	34 (59.6)	< 0.001
Glucocorticoid therapy, n (%)	22 (7.9)	3 (2.0)	3 (4.3)	16 (28.1)	< 0.001
High-flow oxygen, n (%)	47 (16.8)	2 (1.3)	3 (4.3)	42 (73.7)	< 0.001
Invasive mechanical ventilation, n (%)	17 (6.1)	0 (0.0)	0 (0.0)	17 (29.8)	< 0.001
ICU admission, n (%)	17 (6.1)	0 (0.0)	0 (0.0)	17 (29.8)	< 0.001
*Complications*					
Respiratory failure, n (%)	49 (17.5)	2 (1.3)	3 (4.3)	44 (77.2)	< 0.001
ARDS, n (%)	37 (13.2)	1 (0.7)	2 (2.9)	34 (59.6)	< 0.001
Sepsis, n (%)	14 (5.0)	0 (0.0)	0 (0.0)	14 (24.6)	< 0.001
Acute heart injury, n (%)	40 (14.3)	9 (5.9)	11 (15.7)	20 (35.1)	< 0.001
Acute kidney injury, n (%)	26 (9.3)	0 (0.0)	6 (8.6)	20 (35.1)	< 0.001
*Prognosis*					
Discharge, n (%)	267 (95.4)	153 (100.0)	70 (100.0)	44 (77.2)	< 0.001
Death, n (%)	13 (4.6)	0 (0.0)	0 (0.0)	13 (22.8)	< 0.001

Data are n (%), Median [IQR] or mean ± SD. p values comparing patients with COVID-19 in different groups are from χ [2] test, Fisher’s exact test, ANOVA or Mann–Whitney U test. p < 0.05 was considered statistically significant. IQR, interquartile range; BMI, body mass index; CRP, C-reactive protein; hs-TnI, hypersensitive troponin I; CK-MB, creatine kinase muscle-brain; ARDS, acute respiratory distress syndrome; COVID-19, coronavirus disease 2019

During hospitalization, 73 patients developed complications (respiratory failure, 49; ARDS, 37; sepsis, 14; acute heart injury, 40; acute kidney injury, 26), and patients with higher LUS score were more likely to have a higher proportion of these complications. Thirteen patients with high LUS score died, and 267 patients were discharged. Patients with low and moderate LUS score did not die during hospitalization.

After a median follow-up of 14 days [IQR, 10–20 days], 37 patients developed ARDS, and 13 died. All non-surviving patients had ARDS. The clinical data of patients with and without adverse events are listed in Table 2. Patients with adverse events were older and had a significantly higher incidence of comorbidities (including hypertension, diabetes, chronic cardiovascular disease and malignancy), lower LYM% and SO2%, higher levels of CRP, D-dimer, hs-TnI and CK-MB, and lower oxygenation index than patients without adverse events. More patients with adverse events were treated with medicines (antiviral, antibiotic and glucocorticoid) and high-flow oxygen than those without adverse events. Only patients with adverse events received invasive mechanical ventilation (n = 17) therapy and admission to the ICU (n = 17).

**Table 2:** Baseline characteristics of COVID-19 Patients on admission according to the presence of adverse events

Variables	All patients (n = 280)	Non-event (n = 243)	Event (n = 37)	p value
*Demographic data*				
Age, year	55 [40 ~ 65]	52 [38 ~ 61]	71 [63 ~ 79]	< 0.001
Male, n (%)	141 (50.4)	121 (49.8)	20 (54.1)	0.725
BMI, Kg/m [2]	23.11 ± 3.31	23.21 ± 3.27	21.31 ± 2.96	0.129
Temperature, ℃	38.4 [37.8 ~ 38.9]	38.1 [37.8 ~ 38.8]	38.6 [38.0 ~ 39.0]	0.113
Respiratory rate, min^−1^	20 [19 ~ 20]	20 [18 ~ 20]	20 [20 ~ 23]	0.001
SaO2 (%)	98 [97 ~ 99]	99 [98 ~ 99]	97 [95 ~ 99]	< 0.001
*Comorbidities, n (%)*	85 (30.4)	55 (22.6)	25 (67.6)	< 0.001
Hypertension, n (%)	58 (20.7)	42 (17.3)	16 (43.2)	0.001
Diabetes, n (%)	27 (9.6)	14 (5.8)	13 (35.1)	< 0.001
Chronic cardiovascular disease, n (%)	24 (8.6)	11 (4.5)	13 (35.1)	< 0.001
Chronic liver diseases, n (%)	11 (3.9)	8 (3.3)	3 (8.1)	0.166
Malignancy, n (%)	13 (4.6)	6 (2.5)	7 (18.9)	< 0.001
*Laboratory results*				
Lymphocytes %	24.29 ± 10.88	26.92 ± 9.38	13.91 ± 10.24	< 0.001
CRP, mg/L	6.30 [2.00 ~ 40.13]	5.44 [1.48 ~ 27.40]	28.2 [4.2 ~ 85.3]	0.002
D-dimer, ug/L	0.99 [0.37 ~ 3.70]	0.43 [0.26 ~ 1.21]	2.60 [1.06 ~ 7.73]	< 0.001
hs-TnI, ng/L	2.6 [1.4 ~ 2.6]	1.8 [0.9 ~ 4.7]	22.0 [8.6 ~ 197.2]	< 0.001
CK-MB, U/L	0.5 [0.3 ~ 6.0]	0.4 [0.3 ~ 0.6]	9.0 [3.8 ~ 30.5]	< 0.001
PaO_2_:FiO_2_, mmHg				
> 300	246 (87.9)	242 (99.6)	4 (10.8)	< 0.001
200–300	22 (7.9)	1 (0.4)	21 (56.8)	< 0.001
< 200	12 (4.3)	0 (0.0)	12 (32.4)	< 0.001
*Treatments*				
Antiviral therapy, n (%)	82 (29.3)	58 (23.9)	24 (64.9)	< 0.001
Antibiotic therapy, n (%)	78 (27.9)	54 (22.2)	24 (64.9)	< 0.001
Glucocorticoid therapy, n (%)	22 (7.9)	10 (4.1)	12 (32.4)	< 0.001
High-flow oxygen, n (%)	47 (16.8)	11 (4.5)	36 (97.3)	< 0.001
Invasive mechanical ventilation, n (%)	17 (6.1)	0 (0.0)	17 (45.9)	< 0.001
ICU admission, n (%)	17 (6.1)	0 (0.0)	17 (45.9)	< 0.001

Data are n (%) or median [ IQR]. p values comparing patients with COVID-19 in different groups and normal control participants are from χ [2] test, or Mann–Whitney U test. p < 0.05 was considered statistically significant; LUS, lung ultrasonography; COVID-19, coronavirus disease 2019

### LUS characteristics

The median time from admission to LUS examinations was 7 days (interquartile range [IQR] 3–10). In this study, the most common LUS abnormalities in COVID-19 patients were various forms of B-lines (including well-spaced and multiple coalescent B-lines, 75%), followed by pleural line abnormalities (including irregular and blurred pleural line, 46.5%) and lung consolidation (16.4%). Pleural effusion was uncommon. The LUS characteristics of patients with low, moderate and high LUS score are shown in Table 3. Patients with high LUS score were more likely to have bilateral involvement, lung consolidation, pleural line abnormalities, and more B-lines and involved zones. The LUS characteristics of patients with and without adverse events are listed in Table 4. The adverse event group had a higher LUS score (32 vs. 1, p < 0.001) than the non-event group. Patients with adverse outcomes were more likely to have a higher rate of irregular pleural line (97.3% vs. 25.9%, p < 0.001), blurred pleural line (67.6% vs. 2.5%, p < 0.001), multiple coalescent B-lines (70.3% vs. 3.3%, p < 0.001) and lung consolidation (64.9% vs. 9.1%, p < 0.001).

**Table 3:** LUS Characteristics of patients with COVID-19 according to LUS score

Variables	Total population (n = 280)	LUS score			p value
		Low 0–1 (n = 153)	Moderate 2–12 (n = 70)	High > 12 (n = 57)	
LUS features					
Pleural line, n (%)					
Normal pleural line	181 (64.6)	152 (99.3)	29 (41.4)	0 (0.0)	< 0.001
Irregular pleural line	99 (35.4)	1 (0.7)	41 (58.6)	57 (100.0)	< 0.001
Blurred pleural line	31 (11.1)	0 (0.0)	0 (0.0)	31 (54.4)	< 0.001
B-lines, n (%)					
Well-spaced B-lines < 3	250 (89.3)	153 (100.0)	70 (100.0)	27 (47.4)	< 0.001
Well-spaced B-lines ≥ 3	176 (62.9)	49 (32.0)	70 (100.0)	57 (100.0)	< 0.001
Multiple coalescent B-lines	34 (12.1)	0 (0.0)	1 (1.4)	33 (57.9)	< 0.001
Consolidation, n (%)	46 (16.4)	0 (0.0)	3 (4.3)	43 (75.4)	< 0.001
Pleural effusion, n (%)	5 (1.8)	0 (0.0)	0 (0.0)	5 (8.8)	< 0.001
Distribution of abnormal LUS features					
Irregular pleural line					
Anterior fields	55 (19.6)	1 (0.7)	8 (11.4)	46 (80.7)	< 0.001
Lateral fields	50 (17.9)	0 (0.0)	5 (7.1)	45 (78.9)	< 0.001
Posterior fields	76 (27.1)	0 (0.0)	31 (44.3)	45 (78.9)	< 0.001
No. of involved zones	0 [0 ~ 2]	0 [0 ~ 0]	1 [0 ~ 2]	6 [4 ~ 10]	< 0.001
Blurred pleural line					
Anterior fields	4 (1.4)	0 (0.0)	0 (0.0)	4 (7.0)	0.002
Lateral fields	16 (5.7)	0 (0.0)	0 (0.0)	16 (28.1)	< 0.001
Posterior fields	28 (10.0)	0 (0.0)	0 (0.0)	28 (49.1)	< 0.001
No. of involved zones	0 [0 ~ 0]	0 [0 ~ 0]	0 [0 ~ 0]	1 [0 ~ 3]	< 0.001
Well-spaced B-lines ≥ 3					
Anterior fields	100 (35.7)	14 (9.2)	30 (42.9)	56 (98.2)	< 0.001
Lateral fields	109 (38.9)	9 (5.9)	44 (62.9)	56 (98.2)	< 0.001
Posterior fields	139 (49.6)	26 (17.0)	56 (80.0)	57 (100.0)	< 0.001
No. of involved zones	1 [0 ~ 4]	0 [0 ~ 1]	3 [2 ~ 4]	9 [7 ~ 11]	< 0.001
Multiple coalescent B-lines					
Anterior fields	33 (11.8)	0 (0.0)	1 (1.4)	32 (56.1)	< 0.001
Lateral fields	32 (11.4)	0 (0.0)	0 (0.0)	32 (56.1)	< 0.001
Posterior fields	34 (12.1)	0 (0.0)	1 (1.4)	33 (57.9)	< 0.001
No. of involved zones	0 [0 ~ 0]	0 [0 ~ 0]	0 [0 ~ 0]	1 [0 ~ 3]	< 0.001
Consolidation					
Anterior fields	17 (6.1)	0 (0.0)	0 (0.0)	17 (29.8)	< 0.001
Lateral fields	25 (8.9)	0 (0.0)	1 (1.4)	24 (42.1)	< 0.001
Posterior fields	41 (14.6)	0 (0.0)	2 (2.9)	39 (68.4)	< 0.001
No. of involved zones	0 [0 ~ 0]	0 [0 ~ 0]	0 [0 ~ 0]	2 [1 ~ 5]	< 0.001
Left lung involved, n (%)	139 (49.6)	26 (17.0)	56 (80.0)	57 (100.0)	< 0.001
Right lung involved, n (%)	140 (50.0)	23 (15.0)	60 (85.7)	57 (100.0)	< 0.001
Bilateral involved, n (%)	103 (36.8)	0 (0.0)	46 (65.7)	57 (100.0)	< 0.001

Data are n (%) or median [ IQR]. p values comparing patients with COVID-19 in different groups and normal control participants are from χ [2] test, or Mann–Whitney U test. p < 0.05 was considered statistically significant; LUS, lung ultrasonography; COVID-19, coronavirus disease 2019

**Table 4:** LUS Findings of COVID-19 Patients With and Without Adverse Events

Variables	All patients (n = 280)	Non-event (n = 243)	Event (n = 37)	p value
LUS features				
Pleural line, n (%)				
Normal pleural line	181 (64.6)	180 (74.1)	1 (2.7)	< 0.001
Irregular pleural line	99 (35.4)	63 (25.9)	36 (97.3)	< 0.001
Blurred pleural line	31 (11.1)	6 (2.5)	25 (67.6)	< 0.001
B-lines, n (%)				
Well-spaced B-lines < 3	250 (89.3)	236 (97.1)	14 (37.8)	< 0.001
Well-spaced B-lines ≥ 3	176 (62.9)	139 (57.2)	37 (100.0)	< 0.001
Multiple coalescent B-lines	34 (12.1)	8 (3.3)	26 (70.3)	< 0.001
Consolidation, n (%)	46 (16.4)	22 (9.1)	24 (64.9)	< 0.001
Pleural effusion, n (%)	5 (1.8)	3 (1.2)	2 (5.4)	0.131
Distribution of abnormal LUS features				
Irregular pleural line				
Anterior fields	55 (19.6)	25 (10.3)	30 (81.1)	< 0.001
Lateral fields	50 (17.9)	24 (9.9)	26 (70.3)	< 0.001
Posterior fields	76 (27.1)	49 (20.2)	27 (73.0)	< 0.001
No. of involved zones	0 [0 ~ 2]	0 [0 ~ 1]	7 [4 ~ 10]	< 0.001
Blurred pleural line				
Anterior fields	4 (1.4)	1 (0.4)	3 (8.1)	0.008
Lateral fields	16 (5.7)	3 (1.2)	13 (35.1)	< 0.001
Posterior fields	28 (10.0)	6 (2.5)	22 (59.5)	< 0.001
No. of involved zones	0 [0 ~ 0]	0 [0 ~ 0]	2 [0 ~ 3]	< 0.001
Well-spaced B-lines ≥ 3				
Anterior fields	100 (35.7)	63 (25.9)	37 (100.0)	< 0.001
Lateral fields	109 (38.9)	74 (30.5)	35 (94.6)	< 0.001
Posterior fields	139 (49.6)	103 (42.4)	36 (97.3)	< 0.001
No. of involved zones	1 [0 ~ 4]	1 [0 ~ 2]	10 [6 ~ 10]	< 0.001
Multiple coalescent B-lines				
Anterior fields	33 (11.8)	7 (2.9)	26 (70.3)	< 0.001
Lateral fields	32 (11.4)	7 (2.9)	25 (67.6)	< 0.001
Posterior fields	34 (12.1)	8 (3.3)	26 (70.3)	< 0.001
No. of involved zones	0 [0 ~ 0]	0 [0 ~ 0]	2 [0 ~ 3]	< 0.001
Consolidation				
Anterior fields	17 (6.1)	5 (2.1)	12 (32.4)	< 0.001
Lateral fields	25 (8.9)	10 (4.1)	15 (40.5)	< 0.001
Posterior fields	41 (14.6)	19 (7.8)	22 (59.5)	< 0.001
No. of involved zones	0 [0 ~ 0]	0 [0 ~ 0]	2 [0 ~ 5]	< 0.001
Left lung involved, n (%)	139 (49.6)	103 (42.4)	36 (97.3)	< 0.001
Right lung involved, n (%)	140 (50.0)	103 (42.4)	37 (100.0)	< 0.001
Bilateral involved, n (%)	103 (36.8)	67 (27.6)	36 (97.3)	< 0.001
LUS score	1 [0 ~ 6]	1 [0 ~ 3]	32 [21 ~ 49]	< 0.001

Data are n (%) or median [ IQR]. p values comparing patients with COVID-19 in different groups and normal control participants are from χ [2] test, or Mann–Whitney U test. p < 0.05 was considered statistically significant; LUS, lung ultrasonography; COVID-19, coronavirus disease 2019

### Determination of discrimination abilities of independent predictors of adverse outcomes

**Fig. 2:**
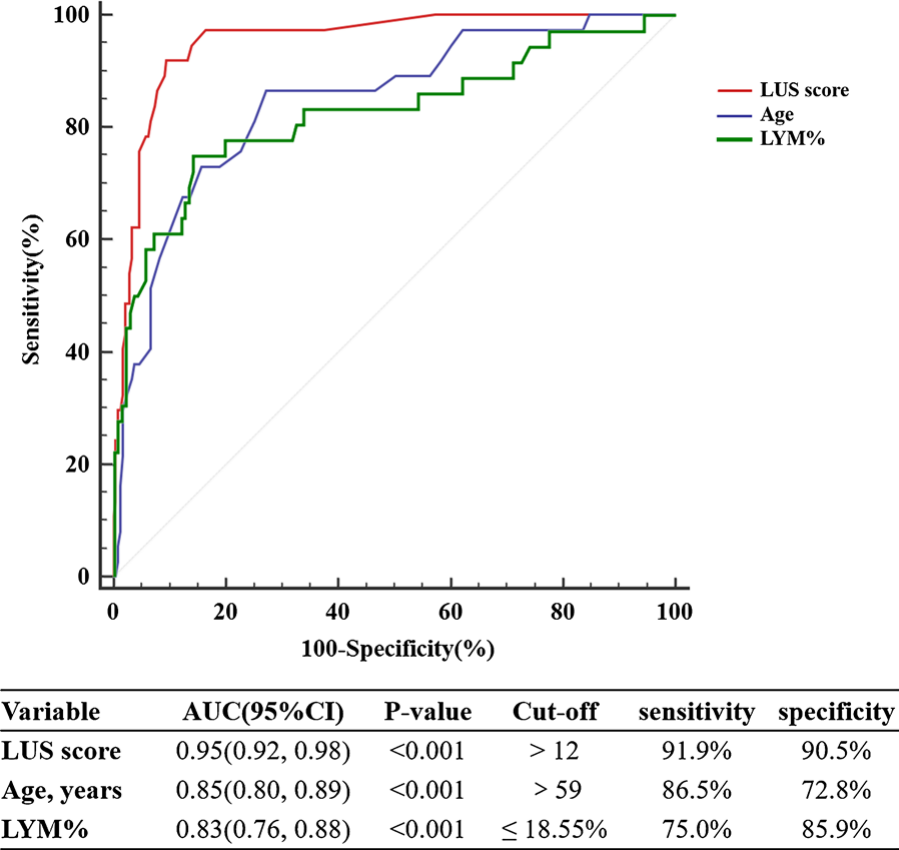
ROC curve analysis for the prediction of adverse events during hospitalization. LYM%, lymphocyte percentage; ROC, receiver operating characteristic

ROC curve analysis was used to assess the predictive values of these three independent predictors (age, LYM%, LUS score) for adverse events during hospitalization. Our results showed that the areas under the curves of LUS score, age and LYM% were 0.95, 0.85 and 0.83, respectively (p < 0.001) (Fig. 2). The area under the curve of the LUS score was greater than that of age (0.95 vs 0.85, p < 0.001) and LYM% (0.95 vs 0.83, p < 0.001). A cut-off value of 12 for the LUS score at admission had a sensitivity of 91.9% and a specificity of 90.5% for the prediction of adverse outcomes in patients with COVID-19.

**Fig. 3:**
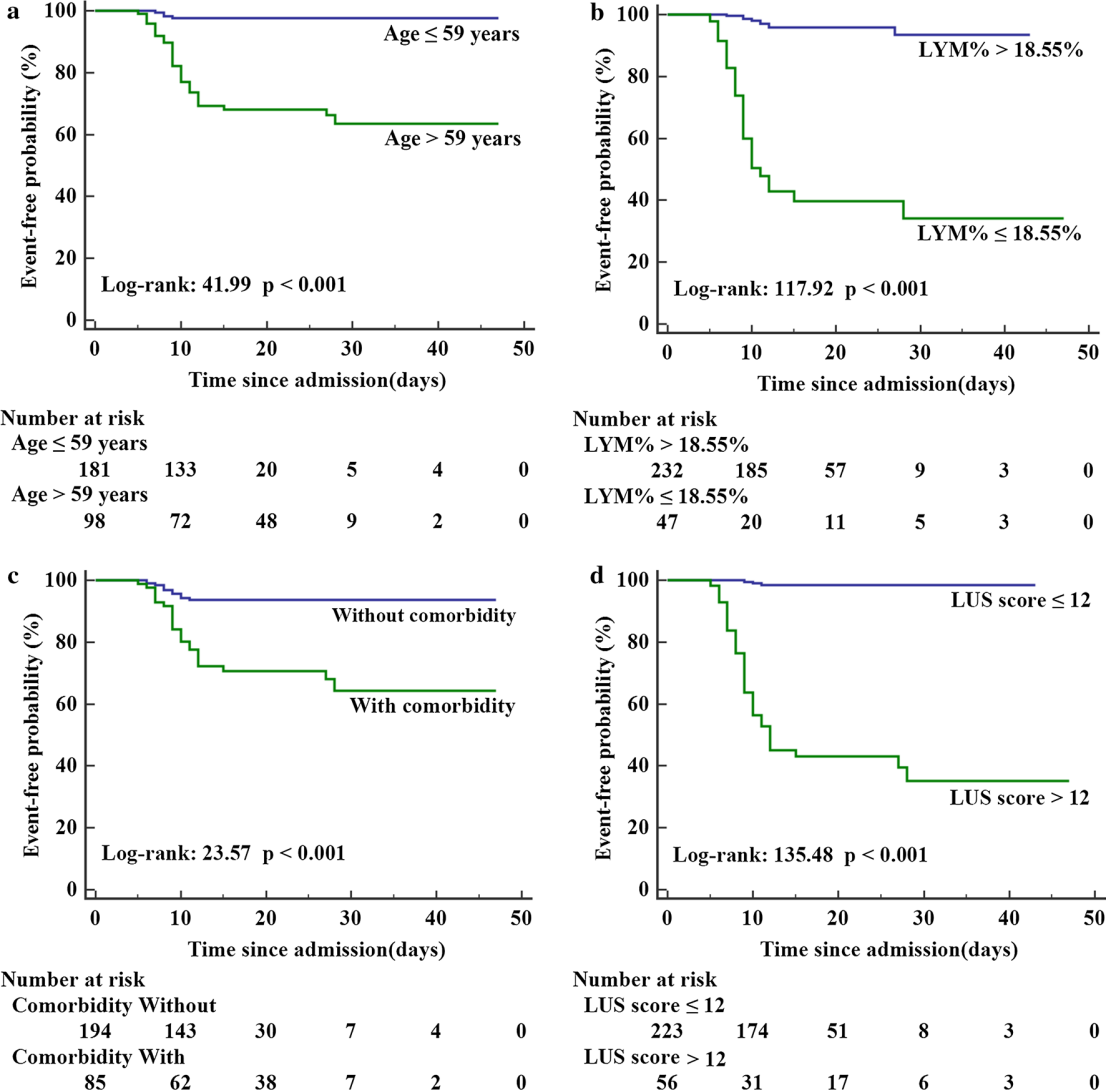
Kaplan–Meier freedom from event curves according to **a** age, **b** lymphocyte percentage (LYM%), **c** comorbidity, and **d** LUS score for the total population

Kaplan–Meier analysis showed that patients with a comorbidity, LUS score > 12, LYM% ≤ 18.55% or age > 59 years were associated with adverse events during hospitalization (Fig. 3).

### Predictors of adverse outcomes in patients with COVID-19

Univariate Cox regression analysis revealed that age (HR 1.081, 95% CI 1.057 ~ 1.106; P < 0.001), LYM% (HR 0.872, 95% CI 0.836 ~ 0.909; P < 0.001), comorbidity (HR 4.928, 95% CI 2.417 ~ 10.050; P < 0.001) and LUS score (HR 1.083, 95% CI 1.065 ~ 1.100; P < 0.001) at admission were significantly associated with adverse events during hospitalization (Table 4). In multivariate Cox analysis models, older age and lower LYM% remained predictive of adverse outcomes; however, the presence of a comorbidity was no longer associated with poor outcomes. The LUS score remained a continuous variable in model 2 and was transformed into a categorical variable according to ROC cut-off points in model 3. The models with clinical parameters and LUS score as a continuous variable (HR 1.049, 95% CI 1.023 ~ 1.078; P < 0.001; AIC = 272; C-index = 0.903) or as a categorical variable (HR 10.76, 95% CI 2.75 ~ 42.05; P = 0.001; AIC = 272; C-index = 0.902) were better in predicting adverse events compared with the basic risk model (age, LYM% and comorbidity) (AIC = 286; C-index = 0.866). (Table 5).

**Table 5:** Predictors of Adverse Event in Patients With COVID-19 by Cox Proportional Hazard Model

Variables	Univariate Cox regression		Model 1 Age + Lymphocytes% + Comorbidity		Model 2 Age + Lymphocytes% + Comorbidity + LUS score		Model 3 Age + Lymphocytes% + Comorbidity + LUS score > 12	
	HR (95% CI)	P value	HR (95% CI)	P value	HR (95% CI)	P value	HR (95% CI)	P value
Age, years	1.081 (1.057, 1.106)	< 0.001	1.040 (1.011, 1.070)	0.007	1.032 (1.001, 1.063)	0.041	1.028 (0.999, 1.059)	0.06
Male (yes vs. no)	1.074 (0.558, 2.068)	0.83						
BMI, Kg/m [2]	0.828 (0.622, 1.103)	0.197						
Lymphocytes%	0.872 (0.836, 0.909)	< 0.001	0.892 (0.853, 0.932)	< 0.001	0.937 (0.892, 0.985)	0.01	0.940 (0.897, 0.985)	0.01
CRP, mg/L	1.007 (1.002, 1.012)	0.01						
hs-TnI, ng/L	1.000 (1.000, 1.001)	0.004						
CK-MB, U/L	1.004 (1.001, 1.008)	0.022						
Comorbidity (yes vs. no)	4.928 (2.417, 10.050)	< 0.001	0.994 (0.453, 2.182)	0.987	0.545 (0.228, 1.299)	0.171	0.695 (0.316, 1.528)	0.366
LUS score	1.083 (1.065, 1.100)	< 0.001			1.049 (1.023, 1.076)	< 0.001		
LUS score > 12 (yes vs. no)	49.935 (15.296, 163.016)	< 0.001					10.760 (2.753, 42.049)	0.001
AIC			286		272		272	
C-index			0.866		0.903		0.902	

P < 0.05 was considered statistically significant. AIC, Akaike information criterion; C-index, concordance index; CK-MB, creatine kinase muscle-brain; CRP, C-reactive protein; hs-TnI, hypersensitive troponin I; CI, confidence interval; HR, hazard ratio

## Discussion

In this study, patients with the highest LUS score were more likely to have higher levels of cardiac injury, coagulopathy and inflammatory biomarkers, more mechanical ventilation therapy, higher incidence of respiratory failure, ARDS, sepsis and higher mortality. Patients with adverse events presented a higher rate of bilateral involvement, more involved zones, B-lines, pleural line abnormalities and consolidation, and a higher LUS score than event-free survivors. More importantly, the LUS score was able to predict a higher risk of adverse events in patients with COVID-19 independently. Therefore, the LUS score may be essential for risk stratification in COVID-19 patients.

Although chest CT has played a crucial role in characterizing pulmonary lesions during the COVID-19 pandemic, the increasing risk of infection and the need to move unstable patients make chest CT a limited choice. The histopathology of pulmonary lesions in COVID-19 patients begins in subpleural regions and is characterized by alveolar damage and oedema, interstitial thickening and consolidation [8]. Furthermore, lesions of this disease are mainly located peripherally and subpleurally [22, 23]. Therefore, ultrasound can identify pulmonary lesions in a timely and sensitive manner. Most patients in our cohort showed bilateral and posterior field involvement, which is consistent with chest CT features [22]. In our study, the predominant LUS abnormality of COVID-19 was B-lines (75%). Patients in our cohort also presented with irregular (35.4%) or blurred (11.1%) pleural line and lung consolidation (16.4%) on LUS. These imaging features characterized in our study are similar to prior studies targeting patients with COVID-19 [24–27].

A previous study showed that the median time from illness onset to ARDS was 12 days (9.5–17.0), and the median time from illness onset to death was 18.5 days (15.0–22.0) [28]. A recent observation regarding the lung changes on chest CT demonstrated that the involvement of lung area and dense consolidation increased to the peak at 9–13 days after symptom onset [29]. In our study, due to the personnel and resource constraints in the early stage of pandemic, we performed LUS examination with some delay. The median time from admission to LUS examinations was 7 (3–10) days, and the median time from illness onset to LUS examinations was 10 days (IQR 5–15). Therefore, we acknowledged that some patients may be at the peak of the disease when performed LUS examinations. In addition, we described serial bedside LUS and corresponding CT findings in a severe (Additional file 1: Fig. 1) and a mild (Additional file 2: Fig. 2) COVID-19 patient to illustrate that performing LUS with some delay allowed the pulmonary lesions and LUS findings to be better developed. In recent studies, CT scans were performed in both the early-phase (within one week) and late-phase (one week later after symptom onset) COVID-19 patients. Their data showed that radiological findings can accurately predict poor outcome irrespective of the disease course [30, 31]. Accordingly, we reckon that the LUS score devised by our group may also have the predictive value in the late-phase patients.

There are several reports regarding lung score. In intensive care units, the most frequently used score distinguishes four steps of progressive loss of aeration, A-lines or two or fewer B-lines (normal aeration, score 0), three or more well-spaced B-lines (moderate loss of aeration, score 1), coalescent B-lines (severe loss of aeration, score 2) and a tissue-like pattern (complete loss of aeration, score 3) [21]. In heart failure patients, the number and spatial extent of B-lines on the antero-lateral chest is usually summed to generate a B-line score to estimate extravascular lung water (EVLW) semi-quantitatively (B-lines ≤ 5, score 0; 6–15, score 1; 16–30, score 2; > 30, score 3) [32]. These lung score, which were based on B-lines, can provide useful information regarding the presence and degree of pulmonary lesions. B-lines are non-specific artefacts associated with increased extravascular lung water or partial loss of lung aeration [20], and they can be detected in a variety of pulmonary diseases, including interstitial lung disease, heart failure, acute respiratory distress syndrome, etc. However, LUS manifestations in COVID-19 patients shared not only the features of an increase in B-lines but also consolidations, irregular or blurred pleural line. The comprehensive assessment of these abnormalities can accurately reflect lung involvement and then serve as a predictor of poor outcomes in patients with COVID-19. Therefore, we proposed the LUS score as an LUS quantitative indicator, which takes into account multiple LUS signs, such as the number of B-lines, consolidation or not, and pleural line changes.

There are limited data regarding the prognostic value of the LUS score in pulmonary disease. In a recent study of 40 elderly patients, Bouhemad et al. found that LUS alone may identify elderly patients at high risk of weaning or extubation failure [33]. Another observation was reported by Platz et al., who demonstrated that pulmonary congestion assessed by ultrasound is associated with other features of clinical congestion and identified those who have a worse prognosis [34]. Similarly, residual pulmonary congestion assessed by a B-line count ≥ 30 is a strong predictor of all-cause death or heart failure hospitalization [35]. These studies employed LUS, which was based on B-lines, for the prediction of pulmonary disease. In our study, we identified that patients with poor outcomes presented a higher rate of bilateral involvement, more involved zones and B-lines, pleural line abnormalities and consolidation, and a higher LUS score. These results revealed that the number of B-lines and the extent of lung consolidation and pleural line abnormality increased with illness severity, suggesting that the LUS score may aid in the classification of disease severity and triage of COVID-19 patients.

COVID-19 can lead to varying degrees of illness, and some patients with mild symptoms at admission may progress rapidly during hospitalization [3]. It is significant to recognize patients with COVID-19 at higher risk for adverse outcomes who might benefit from watchful monitoring. Prior research suggests that patients with COVID-19 who had an older age, lymphopenia, elevated CRP or comorbidity are at higher risk for adverse outcome and death [4–7]. However, quantitative imaging data characterizing the pulmonary lesions would help us to identify patients who are at higher risk of poor outcomes. To the best of our knowledge, this is the first study to assess the prognostic implication of the LUS score in patients with COVID-19. Indeed, patients with a higher LUS score were more likely to experience more adverse clinical events, including mortality or ARDS. Patients with adverse outcomes presented more B-lines, a wider range of pleural line abnormalities and consolidation and a higher LUS score. The LUS score was able to predict a higher risk of adverse outcomes in COVID-19 patients, independent of and incrementally to other clinical parameters. A higher LUS score was not specific for COVID-19-associated lung injury but instead could identify the patients at higher risk for poor outcome.

Several limitations of our study should be highlighted. This was a single-centre study with a relatively limited sample size, which could limit the generalizability of our results. Therefore, further multi-centre studies with a larger sample size are needed to assess the prognostic value of the LUS score in patients with COVID-19. Moreover, LUS can only evaluate peripheral lesions due to echo attenuation, and the actual severity of lung involvement in this cohort may be underestimated. Furthermore, due to the personnel and resource constraints in the early stage of pandemic, we performed LUS examination with some delay, which may limit the prognostic value of LUS score in our study. Additionally, we excluded some patients due to a suboptimal ultrasound window, which might have introduced a bias. Finally, a comparison between the LUS score and chest CT was not performed because we had extremely limited CT image data.

## Conclusions

The LUS score devised by our group performs well at predicting adverse outcomes in patients with COVID-19 and is important for risk stratification in COVID-19 patients.

## Supplementary information

**Additional file 1**. ** Figure 1**: The typical evolution of LUS and corresponding CT findings in a 70-year-old male patient with severe symptom. At day 1, chest CT showed small region of ground-glass opacity (GGO) in the right lower lobe (A1), and LUS was normal (A2); at day 7, the region of GGO was enlarged on CT (B1), and LUS revealed multiple B-lines (B2); at day 11, CT showed bilateral GGO with consolidation in both lower and upper lobes (C1), and LUS demonstrated bilateral consolidation with multiple B-lines (C2). After the second week, the consolidation and GGO were gradually absorbed on chest CT (D1 and E1), LUS demonstrated decreased B-lines, and the consolidation was disappeared (D2 and E2).

**Additional file 2**. ** Figure 2**: The typical evolution of LUS and corresponding CT findings in a 30-year-old female patient with mild symptom. At day 2, chest CT showed small region of ground-glass opacity (GGO) in the left lower lobe (A1), and LUS revealed a small amount of B-lines (A2); at day 10, the previous GGO in the left lower lobe gradually absorbed while many new lesions appeared on CT (B1), and LUS revealed the involved areas of B-lines increased (B2); after the second week, the GGO was continually absorbed on chest CT (C1,D1, E1), and LUS demonstrated B-lines gradually decreased until disappeared (C2,D2, E2).

## Data Availability

All data generated or analyzed during this study are included in this published article.

## Abbreviations

ARDS: Acute respiratory distress syndrome
AIC: Akaike information criterion
ANOVA: Analysis of variance
AUC: Area under curve
BMI: Body mass index
COVID-19: Coronavirus disease 2019
C-index: Concordance index
CK-MB: Creatine kinase muscle-brain
COPD: Chronic obstructive pulmonary disease
CRP: C-reactive protein
CT: Computerized tomography
hs-TnI: Hypersensitive troponin I
HR: Hazard ratio
IQR: Interquartile range
ICU: Intensive care unit
LYM%: Lymphocyte percentage
LUS: Lung ultrasound
SARS-CoV-2: Severe acute respiratory syndrome coronavirus 2
SD: Standard deviation
