# Supplementary figures and images for: Prognostic value of bedside lung ultrasound score in patients with COVID-19
Source: Crit Care. 2020 Dec 22;24:700. doi: 10.1186/s13054-020-03416-1 (PMC7754180; doi:10.1186/s13054-020-03416-1)

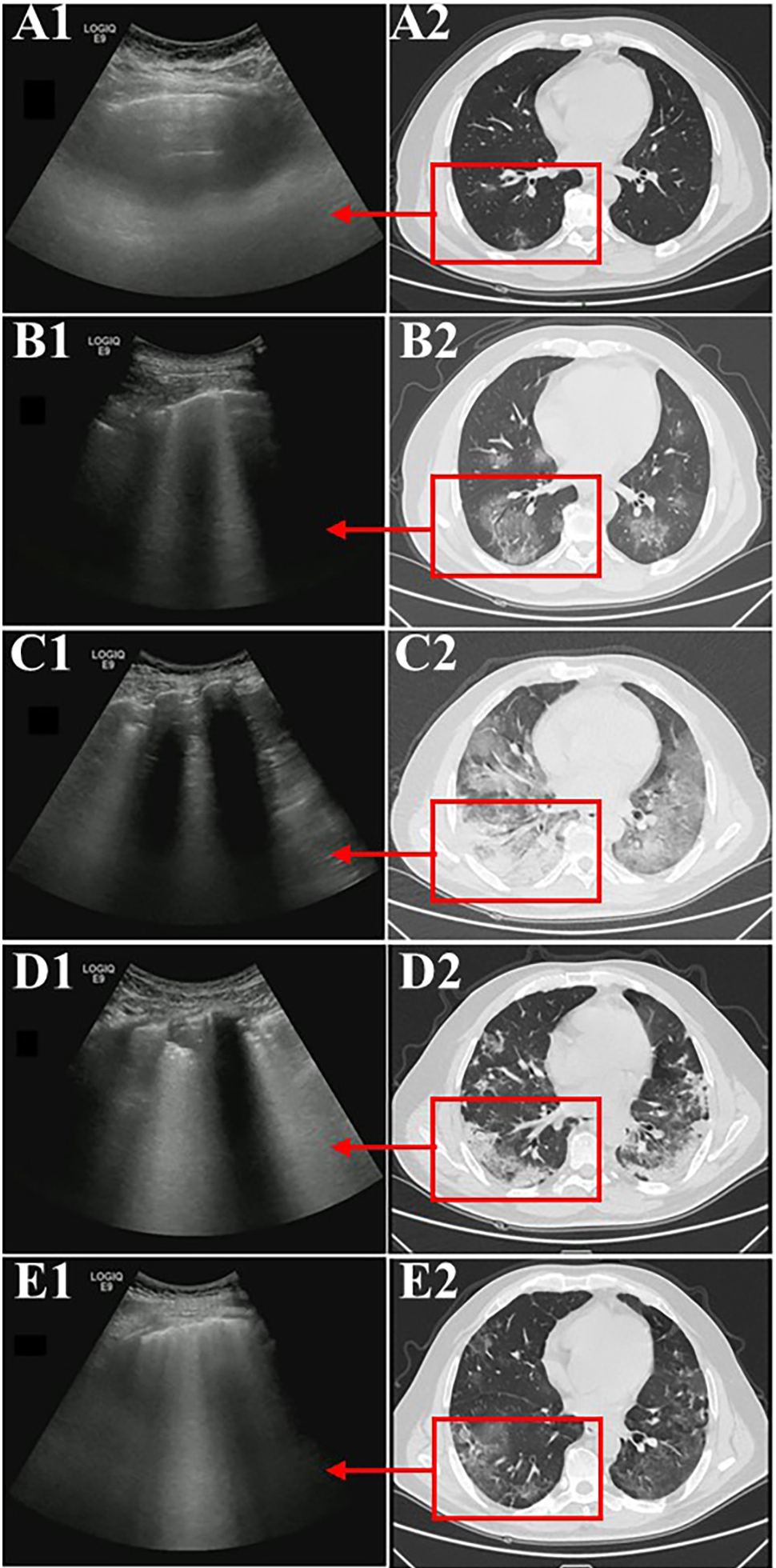

Supplement: Supplementary file 1 — Additional file 1. Figure 1: The typical evolution of LUS and corresponding CT findings in a 70-year-old male patient with severe symptom. At day 1, chest CT showed small region of ground-glass opacity (GGO) in the right lower lobe (A1), and LUS was normal (A2); at day 7, the region of GGO was enlarged on CT (B1), and LUS revealed multiple B-lines (B2); at day 11, CT showed bilateral GGO with consolidation in both lower and upper lobes (C1), and LUS demonstrated bilateral consolidation with multiple B-lines (C2). After the second week, the consolidation and GGO were gradually absorbed on chest CT (D1 and E1), LUS demonstrated decreased B-lines, and the consolidation was disappeared (D2 and E2). [file 13054_2020_3416_MOESM1_ESM.tif]

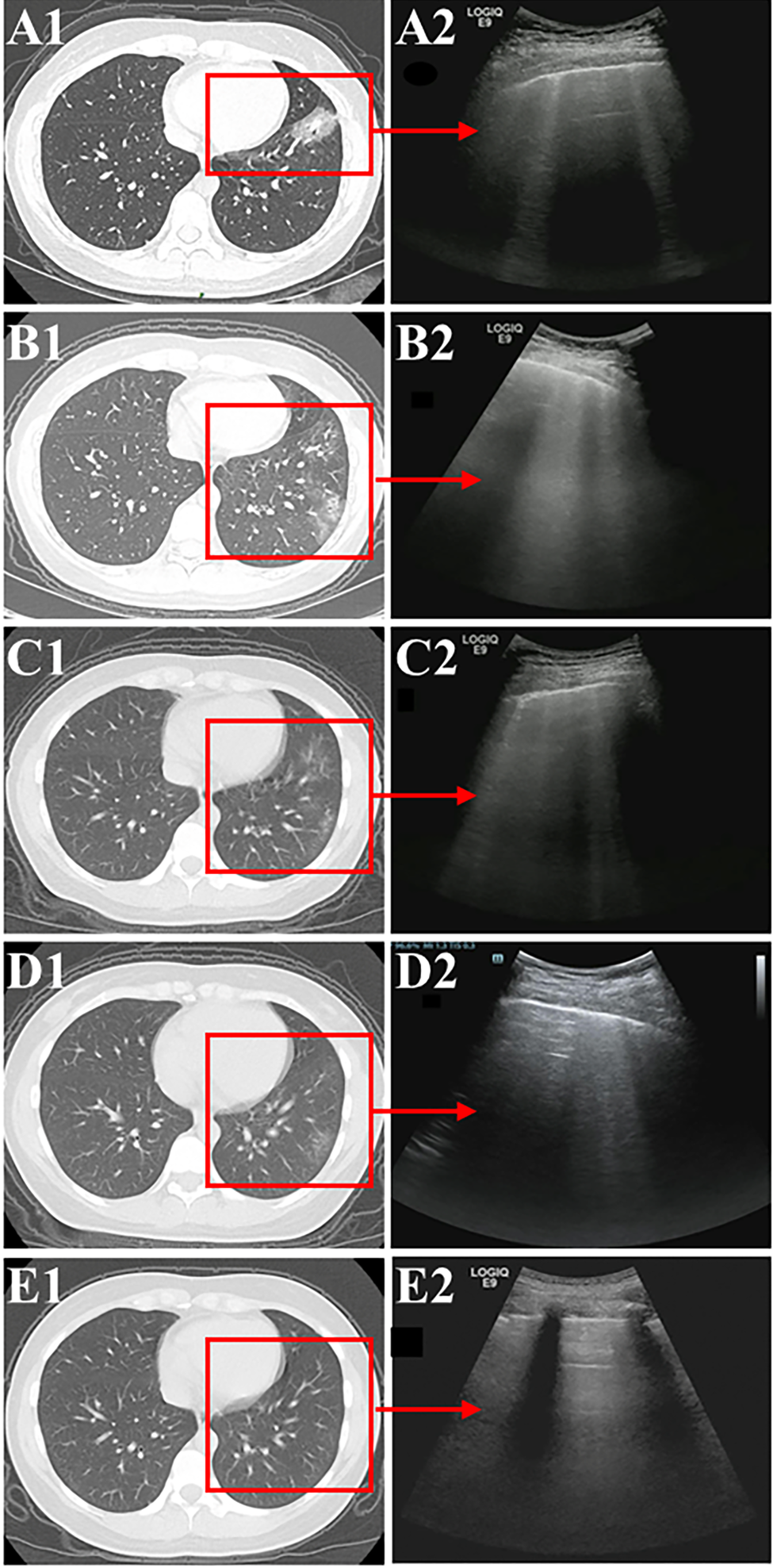

Supplement: Supplementary file 2 — Additional file 2. Figure 2: The typical evolution of LUS and corresponding CT findings in a 30-year-old female patient with mild symptom. At day 2, chest CT showed small region of ground-glass opacity (GGO) in the left lower lobe (A1), and LUS revealed a small amount of B-lines (A2); at day 10, the previous GGO in the left lower lobe gradually absorbed while many new lesions appeared on CT (B1), and LUS revealed the involved areas of B-lines increased (B2); after the second week, the GGO was continually absorbed on chest CT (C1,D1, E1), and LUS demonstrated B-lines gradually decreased until disappeared (C2,D2, E2). [file 13054_2020_3416_MOESM2_ESM.tif]
